# Dicyclo­hexyl­ammonium hydrogen phenyl­phospho­nate

**DOI:** 10.1107/S160053681201553X

**Published:** 2012-04-18

**Authors:** Tidiane Diop, Libasse Diop, Thierry Maris, Helen Stoeckli-Evans

**Affiliations:** a Laboratoire de Chimie Minerale et Analytique, Departement de Chimie, Faculté des Sciences et Techniques, Université Cheikh Anta Diop, Dakar, Senegal; b Departement de Chimie, Université de Montreal, CP 6128, Succ. Centre-Ville, Montreal, Quebec, Canada H3C 3J7; c Institute of Physics, University of Neuchâtel, rue Emile-Argand 11, CH-2000 Neuchâtel, Switzerland

## Abstract

In the title salt, [(C_6_H_11_)_2_NH_2_]^+^·[C_6_H_5_PO_2_(OH)]^−^, the anion is monodeprotonated and acts as both a hydrogen-bond donor and acceptor. The anions are linked by pairs of O—H⋯O inter­actions, forming inversion dimers with *R*_2_^2^(8) ring motifs. These dimers are bridged by two dicyclo­hexyl­aminium cations *via* pairs of N—H⋯O hydrogen bonds, giving *R*_4_^4^(12) ring motifs, forming chains propagating along [010]. The chains are bridged by C—H⋯O inter­actions, forming a two-dimensional network lying parallel to (101).

## Related literature

For the crystal structure of phenyl­phospho­nic acid, see: Weakley (1976 ▶). For the crystal structure of anilinium phenyl­phospho­nate, see: Mahmoudkhani & Langer (2002 ▶). For hydrogen-bond motifs, see: Bernstein *et al.* (1995 ▶).
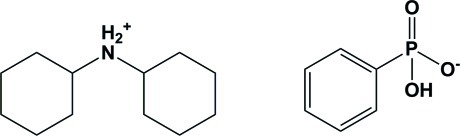


## Experimental

### 

#### Crystal data

C_12_H_24_N^+^·C_6_H_6_O_3_P^−^
*M* *_r_* = 339.40
Monoclinic, 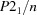

*a* = 13.3212 (4) Å
*b* = 8.9093 (3) Å
*c* = 16.0670 (5) Å
β = 104.385 (1)°
*V* = 1847.09 (10) Å^3^
*Z* = 4
Cu *K*α radiation
μ = 1.43 mm^−1^
*T* = 150 K
0.16 × 0.12 × 0.08 mm

#### Data collection

Bruker Microstar diffractometer
Absorption correction: multi-scan (*SADABS*; Sheldrick, 2004 ▶) *T*_min_ = 0.749, *T*_max_ = 0.892
21802 measured reflections
3456 independent reflections
3221 reflections with *I* > 2σ(*I*)
*R*_int_ = 0.045

#### Refinement

*R*[*F* ^2^ > 2σ(*F* ^2^)] = 0.036
*wR*(*F* ^2^) = 0.098
*S* = 1.08
3456 reflections
210 parameters
H-atom parameters constrained
Δρ_max_ = 0.34 e Å^−3^
Δρ_min_ = −0.34 e Å^−3^

### 

Data collection: *APEX2* (Bruker, 2009 ▶); cell refinement: *SAINT* (Bruker, 2009 ▶); data reduction: *SAINT*; program(s) used to solve structure: *SHELXS97* (Sheldrick, 2008 ▶); program(s) used to refine structure: *SHELXL97* (Sheldrick, 2008 ▶); molecular graphics: *SHELXTL* (Sheldrick, 2008 ▶) and *Mercury* (Macrae *et al.*, 2008 ▶); software used to prepare material for publication: *SHELXTL* and *publCIF* (Westrip, 2010 ▶).

## Supplementary Material

Crystal structure: contains datablock(s) I, global. DOI: 10.1107/S160053681201553X/hb6721sup1.cif

Structure factors: contains datablock(s) I. DOI: 10.1107/S160053681201553X/hb6721Isup2.hkl

Supplementary material file. DOI: 10.1107/S160053681201553X/hb6721Isup3.cml

Additional supplementary materials:  crystallographic information; 3D view; checkCIF report

## Figures and Tables

**Table 1:** Hydrogen-bond geometry (Å, °)

*D*—H⋯*A*	*D*—H	H⋯*A*	*D*⋯*A*	*D*—H⋯*A*
O3—H3*A*⋯O1^i^	0.84	1.75	2.5920 (13)	175
N1—H1*A*⋯O1	0.92	1.86	2.7520 (14)	161
N1—H1*B*⋯O2^ii^	0.92	1.81	2.6897 (15)	159
C18—H18*A*⋯O3^iii^	0.99	2.52	3.3019 (16)	136
C18—H18*B*⋯O2^ii^	0.99	2.52	3.2693 (16)	133

Symmetry codes: (i) 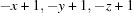; (ii) 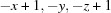; (iii) 
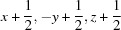
.

**Fig. 1.**
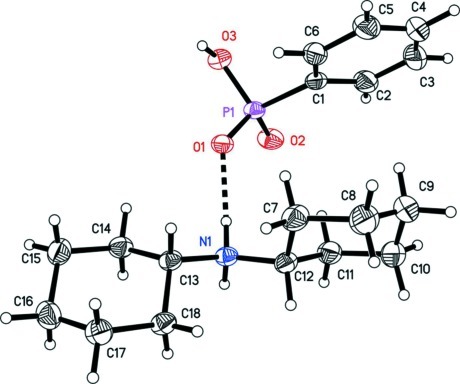
The molecular structure of the title salt. Displacement ellipsoids are drawn at the 50% probability level [an N-H···O hydrogen bond is shown as a dashed line].

**Fig. 2.**
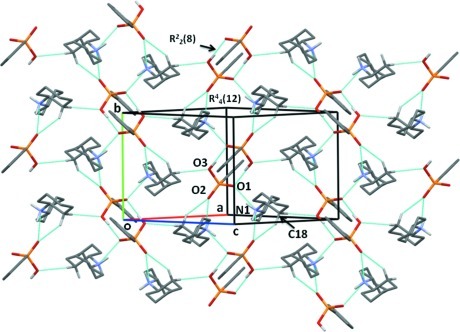
A view of the crystal packing of the title salt. The O-H···O and N-H···O hydrogen bonds, and the C-H···O interactions are shown as dashed cyan lines (see Table 1 for details; H atoms not involved in these interactions have been omitted for clarity).

## supplementary crystallographic information

### Comment

In the title salt (Fig. 1), the hydrogen phenylphosphonate anion is unequivocally tetrahedral, with three oxygen atoms and a phenyl group; O2—P1—O1 116.59 (5)°, O3—P1—C1 104.45 (5)°. The P—O distances are different [1.4870 (10) Å for P═O, 1.5134 (9) Å for P-O^-^, and 1.574 (3) Å for bond P-O(H)]. This is similar to the situation in the crystal structure of the parent phenylphosphonic acid PhPO_3_H_2_ (Weakley, 1976), with bond distances of 1.496 Å for P═O and 1.545 Å for P—O(H).

In the crystal, the anions are linked by a pair of O-H···O hydrogen bonds to form inversion dimers with a ring motif of R^2^_2_(8) (Bernstein *et al.*, 1995; Table 1 and Fig. 2). These dimers are linked by two dicyclohexylaminium cations, via pairs of N-H···O hydrogen bonds forming a ring motif of R^4^_4_(12), to form chains propagating along [010], as shown in Table 1 and Fig. 2. A similar chain arrangement has been observed in the crystal structure of anilinium phenylphosphonate (Mahmoudkhani & Langer, 2002). In the title compound the chains are linked by C-H···O interactions to form a two-dimensional network that lies parallel to (101); see Table 1 and Fig. 2.

### Experimental

When dicyclohexylamine was allowed to react with phenylphosphonic acid in an equimolar ratio (1:1) in water, a precipitate was obtained and filtered [Yield: 83%; *M*.p: 448 K]. The filtrate was allowed to evaporate at 333 K, giving colourless block-like crystals of the title compound.

### Refinement

The OH and NH2 H atoms could be located in a difference electron density map. For refinement all the H-atoms were placed in calculated positions and treated as riding atoms: O-H = 0.84 Å, N-H = 0.92 Å, C-H = 0.95, 0.99 and 1.00 Å for CH(aromatic), methylene and methine H atoms, respectively, with U_iso_ = k × U_eq_(O,N,C), where k = 1.5 for OH and NH_2_ H atoms, and = 1.2 for other H atoms.

### Crystal data

**Table:**

C_12_H_24_N^+^·C_6_H_6_O_3_P^−^	*F*(000) = 736
*M**_r_* = 339.40	*D*_x_ = 1.220 Mg m^−^^3^
Monoclinic, *P*2_1_/*n*	Cu *K*α radiation, λ = 1.54178 Å
Hall symbol: -P 2yn	Cell parameters from 10987 reflections
*a* = 13.3212 (4) Å	θ = 3.9–69.5°
*b* = 8.9093 (3) Å	µ = 1.43 mm^−^^1^
*c* = 16.0670 (5) Å	*T* = 150 K
β = 104.385 (1)°	Block, colourless
*V* = 1847.09 (10) Å^3^	0.16 × 0.12 × 0.08 mm
*Z* = 4	

### Data collection

**Table:**

Bruker Microstar diffractometer	3456 independent reflections
Radiation source: Rotating Anode	3221 reflections with *I* > 2σ(*I*)
Helios optics monochromator	*R*_int_ = 0.045
Detector resolution: 8.3 pixels mm^-1^	θ_max_ = 70.0°, θ_min_ = 3.9°
ω scans	*h* = −15→16
Absorption correction: multi-scan (*SADABS*; Sheldrick, 2004)	*k* = −10→10
*T*_min_ = 0.749, *T*_max_ = 0.892	*l* = −19→17
21802 measured reflections	

### Refinement

**Table:**

Refinement on *F*^2^	Primary atom site location: structure-invariant direct methods
Least-squares matrix: full	Secondary atom site location: difference Fourier map
*R*[*F*^2^ > 2σ(*F*^2^)] = 0.036	Hydrogen site location: inferred from neighbouring sites
*wR*(*F*^2^) = 0.098	H-atom parameters constrained
*S* = 1.08	*w* = 1/[σ^2^(*F*_o_^2^) + (0.0485*P*)^2^ + 0.5837*P*] where *P* = (*F*_o_^2^ + 2*F*_c_^2^)/3
3456 reflections	(Δ/σ)_max_ < 0.001
210 parameters	Δρ_max_ = 0.34 e Å^−^^3^
0 restraints	Δρ_min_ = −0.34 e Å^−^^3^

### Special details

**Table:**

Geometry. Bond distances, angles etc. have been calculated using the rounded fractional coordinates. All su's are estimated from the variances of the (full) variance-covariance matrix. The cell esds are taken into account in the estimation of distances, angles and torsion angles
Refinement. Refinement of *F*^2^ against ALL reflections. The weighted *R*-factor *wR* and goodness of fit *S* are based on *F*^2^, conventional *R*-factors *R* are based on *F*, with *F* set to zero for negative *F*^2^. The threshold expression of *F*^2^ > σ(*F*^2^) is used only for calculating *R*-factors(gt) *etc*. and is not relevant to the choice of reflections for refinement. *R*-factors based on *F*^2^ are statistically about twice as large as those based on *F*, and *R*- factors based on ALL data will be even larger.

### Fractional atomic coordinates and isotropic or equivalent isotropic displacement parameters (Å^2^)

**Table:**

	*x*	*y*	*z*	*U*_iso_*/*U*_eq_	
N1	0.61697 (8)	0.08819 (12)	0.61698 (7)	0.0241 (3)	
C7	0.78359 (11)	0.20505 (17)	0.60436 (11)	0.0360 (4)	
C8	0.88185 (13)	0.17940 (19)	0.57279 (13)	0.0474 (6)	
C9	0.85691 (14)	0.1191 (2)	0.48160 (12)	0.0504 (6)	
C10	0.79476 (14)	−0.02535 (19)	0.47509 (11)	0.0456 (6)	
C11	0.69609 (12)	−0.00267 (17)	0.50547 (9)	0.0347 (4)	
C12	0.71952 (10)	0.06220 (15)	0.59617 (9)	0.0270 (4)	
C13	0.61555 (10)	0.16079 (14)	0.70110 (8)	0.0246 (4)	
C14	0.50257 (10)	0.18592 (16)	0.70178 (9)	0.0297 (4)	
C15	0.49544 (11)	0.25630 (18)	0.78668 (10)	0.0359 (4)	
C16	0.54933 (12)	0.15792 (18)	0.86212 (10)	0.0375 (5)	
C17	0.66214 (11)	0.13042 (17)	0.86139 (9)	0.0335 (4)	
C18	0.67143 (10)	0.06407 (15)	0.77601 (8)	0.0285 (4)	
P1	0.48868 (2)	0.32919 (3)	0.40858 (2)	0.0235 (1)	
O1	0.52613 (7)	0.31140 (10)	0.50502 (6)	0.0267 (3)	
O2	0.43638 (8)	0.19710 (11)	0.35992 (6)	0.0353 (3)	
O3	0.41421 (7)	0.46904 (10)	0.38831 (6)	0.0278 (3)	
C1	0.59865 (10)	0.38079 (15)	0.36779 (8)	0.0267 (4)	
C2	0.61401 (14)	0.31648 (17)	0.29338 (10)	0.0401 (5)	
C3	0.69755 (16)	0.3597 (2)	0.26150 (12)	0.0534 (7)	
C4	0.76585 (14)	0.46728 (19)	0.30332 (13)	0.0492 (6)	
C5	0.75229 (12)	0.53178 (18)	0.37773 (11)	0.0401 (5)	
C6	0.66913 (11)	0.48883 (16)	0.40993 (9)	0.0309 (4)	
H1A	0.57730	0.14630	0.57380	0.0360*	
H1B	0.58430	−0.00320	0.61490	0.0360*	
H7A	0.80310	0.23750	0.66520	0.0430*	
H7B	0.74170	0.28580	0.57010	0.0430*	
H8A	0.91990	0.27540	0.57510	0.0570*	
H8B	0.92740	0.10730	0.61150	0.0570*	
H9A	0.92210	0.09980	0.46440	0.0600*	
H9B	0.81670	0.19490	0.44190	0.0600*	
H10A	0.83780	−0.10390	0.51050	0.0550*	
H10B	0.77630	−0.06020	0.41470	0.0550*	
H11A	0.66020	−0.10020	0.50450	0.0420*	
H11B	0.64910	0.06620	0.46550	0.0420*	
H12	0.75910	−0.01410	0.63710	0.0320*	
H13	0.65120	0.26030	0.70480	0.0290*	
H14A	0.46530	0.08880	0.69370	0.0360*	
H14B	0.46920	0.25290	0.65370	0.0360*	
H15A	0.52820	0.35680	0.79260	0.0430*	
H15B	0.42170	0.26900	0.78700	0.0430*	
H16A	0.51270	0.06060	0.85890	0.0450*	
H16B	0.54650	0.20750	0.91660	0.0450*	
H17A	0.69400	0.06080	0.90860	0.0400*	
H17B	0.70060	0.22640	0.87170	0.0400*	
H18A	0.74550	0.05610	0.77590	0.0340*	
H18B	0.64150	−0.03820	0.76940	0.0340*	
H2	0.56710	0.24240	0.26400	0.0480*	
H3	0.70750	0.31480	0.21050	0.0640*	
H3A	0.43610	0.53690	0.42460	0.0420*	
H4	0.82240	0.49710	0.28090	0.0590*	
H5	0.79970	0.60540	0.40690	0.0480*	
H6	0.66000	0.53350	0.46130	0.0370*	

### Atomic displacement parameters (Å^2^)

**Table:**

	*U*^11^	*U*^22^	*U*^33^	*U*^12^	*U*^13^	*U*^23^
N1	0.0276 (5)	0.0192 (5)	0.0238 (5)	−0.0008 (4)	0.0032 (4)	0.0000 (4)
C7	0.0332 (7)	0.0271 (7)	0.0489 (9)	−0.0022 (6)	0.0124 (7)	−0.0015 (6)
C8	0.0360 (8)	0.0408 (10)	0.0700 (12)	−0.0010 (7)	0.0217 (8)	0.0067 (8)
C9	0.0545 (10)	0.0475 (10)	0.0595 (11)	0.0129 (8)	0.0339 (9)	0.0174 (8)
C10	0.0634 (11)	0.0403 (9)	0.0396 (9)	0.0131 (8)	0.0251 (8)	0.0038 (7)
C11	0.0468 (8)	0.0285 (7)	0.0297 (7)	0.0028 (6)	0.0111 (6)	−0.0007 (6)
C12	0.0302 (7)	0.0217 (6)	0.0292 (7)	0.0024 (5)	0.0074 (5)	0.0004 (5)
C13	0.0286 (7)	0.0185 (6)	0.0254 (7)	−0.0005 (5)	0.0044 (5)	−0.0015 (5)
C14	0.0285 (7)	0.0290 (7)	0.0302 (7)	0.0033 (5)	0.0046 (6)	0.0043 (5)
C15	0.0349 (7)	0.0358 (8)	0.0385 (8)	0.0055 (6)	0.0121 (6)	−0.0022 (6)
C16	0.0432 (8)	0.0409 (9)	0.0292 (8)	−0.0010 (7)	0.0105 (6)	−0.0028 (6)
C17	0.0391 (8)	0.0326 (8)	0.0256 (7)	0.0007 (6)	0.0018 (6)	−0.0028 (6)
C18	0.0316 (7)	0.0257 (7)	0.0245 (7)	0.0035 (5)	0.0001 (5)	−0.0016 (5)
P1	0.0288 (2)	0.0179 (2)	0.0229 (2)	−0.0024 (1)	0.0046 (1)	0.0004 (1)
O1	0.0342 (5)	0.0218 (5)	0.0240 (5)	0.0016 (4)	0.0072 (4)	0.0029 (3)
O2	0.0477 (6)	0.0236 (5)	0.0325 (5)	−0.0097 (4)	0.0060 (5)	−0.0035 (4)
O3	0.0270 (5)	0.0248 (5)	0.0285 (5)	0.0002 (4)	0.0008 (4)	0.0000 (4)
C1	0.0343 (7)	0.0201 (6)	0.0258 (7)	0.0042 (5)	0.0079 (5)	0.0043 (5)
C2	0.0615 (10)	0.0278 (8)	0.0359 (8)	−0.0015 (7)	0.0216 (7)	−0.0011 (6)
C3	0.0848 (14)	0.0386 (9)	0.0523 (11)	0.0060 (9)	0.0464 (10)	0.0020 (8)
C4	0.0544 (10)	0.0389 (9)	0.0673 (12)	0.0076 (8)	0.0396 (9)	0.0146 (8)
C5	0.0342 (7)	0.0362 (8)	0.0523 (10)	−0.0008 (6)	0.0151 (7)	0.0093 (7)
C6	0.0313 (7)	0.0299 (7)	0.0318 (7)	0.0003 (6)	0.0084 (6)	0.0036 (6)

### Geometric parameters (Å, º)

**Table:**

P1—O1	1.5136 (10)	C10—H10A	0.9900
P1—O2	1.4869 (10)	C10—H10B	0.9900
P1—O3	1.5755 (10)	C11—H11A	0.9900
P1—C1	1.8066 (14)	C11—H11B	0.9900
O3—H3A	0.8400	C12—H12	1.0000
N1—C12	1.5029 (18)	C13—H13	1.0000
N1—C13	1.5027 (17)	C14—H14B	0.9900
N1—H1A	0.9200	C14—H14A	0.9900
N1—H1B	0.9200	C15—H15A	0.9900
C7—C12	1.520 (2)	C15—H15B	0.9900
C7—C8	1.534 (2)	C16—H16A	0.9900
C8—C9	1.518 (3)	C16—H16B	0.9900
C9—C10	1.520 (3)	C17—H17A	0.9900
C10—C11	1.525 (2)	C17—H17B	0.9900
C11—C12	1.526 (2)	C18—H18B	0.9900
C13—C14	1.5243 (19)	C18—H18A	0.9900
C13—C18	1.5166 (18)	C1—C6	1.397 (2)
C14—C15	1.526 (2)	C1—C2	1.386 (2)
C15—C16	1.523 (2)	C2—C3	1.390 (3)
C16—C17	1.526 (2)	C3—C4	1.376 (3)
C17—C18	1.5267 (19)	C4—C5	1.378 (3)
C7—H7A	0.9900	C5—C6	1.388 (2)
C7—H7B	0.9900	C2—H2	0.9500
C8—H8B	0.9900	C3—H3	0.9500
C8—H8A	0.9900	C4—H4	0.9500
C9—H9A	0.9900	C5—H5	0.9500
C9—H9B	0.9900	C6—H6	0.9500
			
O1—P1—O2	116.59 (5)	C12—C11—H11B	109.00
O1—P1—O3	108.96 (5)	H11A—C11—H11B	108.00
O1—P1—C1	107.90 (6)	N1—C12—H12	109.00
O2—P1—O3	109.18 (6)	C11—C12—H12	109.00
O2—P1—C1	109.08 (6)	C7—C12—H12	109.00
O3—P1—C1	104.45 (6)	C18—C13—H13	109.00
P1—O3—H3A	109.00	N1—C13—H13	109.00
C12—N1—C13	118.80 (10)	C14—C13—H13	109.00
H1A—N1—H1B	107.00	C15—C14—H14A	110.00
C12—N1—H1A	108.00	C13—C14—H14B	110.00
C12—N1—H1B	108.00	C13—C14—H14A	110.00
C13—N1—H1B	108.00	C15—C14—H14B	110.00
C13—N1—H1A	108.00	H14A—C14—H14B	108.00
C8—C7—C12	110.71 (13)	H15A—C15—H15B	108.00
C7—C8—C9	111.83 (15)	C14—C15—H15B	110.00
C8—C9—C10	110.55 (15)	C16—C15—H15A	110.00
C9—C10—C11	111.34 (14)	C14—C15—H15A	109.00
C10—C11—C12	111.58 (13)	C16—C15—H15B	109.00
C7—C12—C11	112.15 (12)	C15—C16—H16B	109.00
N1—C12—C11	106.81 (11)	C15—C16—H16A	109.00
N1—C12—C7	111.94 (11)	H16A—C16—H16B	108.00
C14—C13—C18	111.63 (11)	C17—C16—H16A	109.00
N1—C13—C14	107.62 (10)	C17—C16—H16B	109.00
N1—C13—C18	110.85 (10)	C16—C17—H17B	109.00
C13—C14—C15	110.35 (11)	C16—C17—H17A	109.00
C14—C15—C16	110.69 (13)	C18—C17—H17A	109.00
C15—C16—C17	110.90 (13)	C18—C17—H17B	109.00
C16—C17—C18	111.65 (12)	H17A—C17—H17B	108.00
C13—C18—C17	111.08 (11)	C13—C18—H18A	109.00
C8—C7—H7B	110.00	C13—C18—H18B	109.00
C12—C7—H7A	110.00	C17—C18—H18A	109.00
C12—C7—H7B	109.00	C17—C18—H18B	109.00
C8—C7—H7A	109.00	H18A—C18—H18B	108.00
H7A—C7—H7B	108.00	P1—C1—C2	120.96 (11)
H8A—C8—H8B	108.00	C2—C1—C6	118.53 (13)
C7—C8—H8A	109.00	P1—C1—C6	120.50 (10)
C7—C8—H8B	109.00	C1—C2—C3	120.44 (15)
C9—C8—H8B	109.00	C2—C3—C4	120.38 (17)
C9—C8—H8A	109.00	C3—C4—C5	120.04 (18)
C8—C9—H9B	110.00	C4—C5—C6	119.81 (15)
C8—C9—H9A	110.00	C1—C6—C5	120.80 (13)
H9A—C9—H9B	108.00	C1—C2—H2	120.00
C10—C9—H9A	110.00	C3—C2—H2	120.00
C10—C9—H9B	110.00	C2—C3—H3	120.00
C9—C10—H10B	109.00	C4—C3—H3	120.00
C9—C10—H10A	109.00	C3—C4—H4	120.00
C11—C10—H10A	109.00	C5—C4—H4	120.00
C11—C10—H10B	109.00	C4—C5—H5	120.00
H10A—C10—H10B	108.00	C6—C5—H5	120.00
C10—C11—H11A	109.00	C1—C6—H6	120.00
C10—C11—H11B	109.00	C5—C6—H6	120.00
C12—C11—H11A	109.00		
			
O3—P1—C1—C2	−106.84 (12)	C10—C11—C12—N1	176.67 (12)
O1—P1—C1—C6	−43.88 (13)	N1—C13—C14—C15	178.67 (11)
O2—P1—C1—C6	−171.43 (11)	C14—C13—C18—C17	−55.08 (15)
O3—P1—C1—C6	71.95 (12)	C18—C13—C14—C15	56.83 (15)
O1—P1—C1—C2	137.33 (12)	N1—C13—C18—C17	−175.05 (11)
O2—P1—C1—C2	9.78 (14)	C13—C14—C15—C16	−57.40 (16)
C12—N1—C13—C18	−62.64 (14)	C14—C15—C16—C17	56.75 (16)
C13—N1—C12—C11	−176.40 (11)	C15—C16—C17—C18	−55.13 (16)
C12—N1—C13—C14	175.04 (11)	C16—C17—C18—C13	54.13 (16)
C13—N1—C12—C7	−53.28 (15)	P1—C1—C2—C3	178.46 (13)
C12—C7—C8—C9	55.25 (18)	C6—C1—C2—C3	−0.4 (2)
C8—C7—C12—N1	−173.44 (13)	P1—C1—C6—C5	−178.34 (12)
C8—C7—C12—C11	−53.39 (17)	C2—C1—C6—C5	0.5 (2)
C7—C8—C9—C10	−56.75 (19)	C1—C2—C3—C4	−0.2 (3)
C8—C9—C10—C11	56.27 (19)	C2—C3—C4—C5	0.7 (3)
C9—C10—C11—C12	−54.83 (17)	C3—C4—C5—C6	−0.5 (3)
C10—C11—C12—C7	53.68 (16)	C4—C5—C6—C1	0.0 (2)

### Hydrogen-bond geometry (Å, º)

**Table:**

*D*—H···*A*	*D*—H	H···*A*	*D*···*A*	*D*—H···*A*
O3—H3*A*···O1^i^	0.84	1.75	2.5920 (13)	175
N1—H1*A*···O1	0.92	1.86	2.7520 (14)	161
N1—H1*B*···O2^ii^	0.92	1.81	2.6897 (15)	159
C18—H18*A*···O3^iii^	0.99	2.52	3.3019 (16)	136
C18—H18*B*···O2^ii^	0.99	2.52	3.2693 (16)	133

Symmetry codes: (i) −*x*+1, −*y*+1, −*z*+1; (ii) −*x*+1, −*y*, −*z*+1; (iii) *x*+1/2, −*y*+1/2, *z*+1/2.
